# A SUCCESSFUL MODEL FOR AMPLIFYING THE VOICES OF OLDER ADULTS IN LONG-TERM CARE AND SERVICES AND SUPPORT RESEARCH

**DOI:** 10.1093/geroni/igac059.1452

**Published:** 2022-12-20

**Authors:** Rachel Lessem, Rebecca Berman, Amy Eisenstein

**Affiliations:** CJE SeniorLife, Chicago, Illinois, United States; Leonard Schanfield Research Institute, Chicago, Illinois, United States; RRF Foundation for Aging, Chicago, Illinois, United States

## Abstract

The experiential voice of older adults receiving long term services and supports (LTSS) is largely absent from health research hampering the development of effective interventions. While many have the capacity to participate in the design, development, and delivery of research, researchers traditionally do not recognize such capacity, may be unsure how to seek input, or may not appreciate the extent to which such input can improve the research enterprise. But the participation of these older adults can ensure that patient-centered research meaningfully addresses their care preferences and desired health outcomes, and can improve the effectiveness of care and patients’ quality of life. Through three PCORI-funded projects, we developed a successful model for addressing barriers to amplify these voices. We outline barriers to the dissemination and implementation of this model, and suggest next steps to test strategies to amplify the voices of older adults in long-term care.

